# Effects of Phosphate Shortage on Root Growth and Hormone Content of Barley Depend on Capacity of the Roots to Accumulate ABA

**DOI:** 10.3390/plants9121722

**Published:** 2020-12-07

**Authors:** Lidiya Vysotskaya, Guzel Akhiyarova, Arina Feoktistova, Zarina Akhtyamova, Alla Korobova, Igor Ivanov, Ian Dodd, Bulat Kuluev, Guzel Kudoyarova

**Affiliations:** 1 Ufa Institute of Biology of Ufa Federal Research Centre of the Russian Academy of Sciences, Pr. Octyabrya, 69, 450054 Ufa, Russia; vysotskaya@anrb.ru (L.V.); akhiyarova@rambler.ru (G.A.); feoktistova.arisha@yandex.ru (A.F.); akhtyamovazarina@gmail.com (Z.A.); muksin@mail.ru (A.K.); i_ivanov@anrb.ru (I.I.); 2 The Lancaster Environment Centre, Lancaster University, Lancaster LA1 4YQ, UK; i.dodd@lancaster.ac.uk; 3 Institute of Biochemistry and Genetics of Ufa Federal Research Centre of Russian Academy of Sciences, Pr. Octyabrya, 71, 450054 Ufa, Russia; kuluev@bk.ru; 4 Department of Biology, Bashkir State University, Zaki-Validi St. 32, 450074 Ufa, Russia

**Keywords:** *Hordeum vulgare*, phosphate starvation, ABA-deficient mutant, auxins, cytokinins, root growth

## Abstract

Although changes in root architecture in response to the environment can optimize mineral and water nutrient uptake, mechanisms regulating these changes are not well-understood. We investigated whether P deprivation effects on root development are mediated by abscisic acid (ABA) and its interactions with other hormones. The ABA-deficient barley mutant *Az34* and its wild-type (WT) were grown in P-deprived and P-replete conditions, and hormones were measured in whole roots and root tips. Although P deprivation decreased growth in shoot mass similarly in both genotypes, only the WT increased primary root length and number of lateral roots. The effect was accompanied by ABA accumulation in root tips, a response not seen in *Az34*. Increased ABA in P-deprived WT was accompanied by decreased concentrations of cytokinin, an inhibitor of root extension. Furthermore, P-deficiency in the WT increased auxin concentration in whole root systems in association with increased root branching. In the ABA-deficient mutant, P-starvation failed to stimulate root elongation or promote branching, and there was no decline in cytokinin and no increase in auxin. The results demonstrate ABA’s ability to mediate in root growth responses to P starvation in barley, an effect linked to its effects on cytokinin and auxin concentrations.

## 1. Introduction

Deficiencies in mineral nutrients reduce plant growth and crop yields. Changes in root architecture are an important adaptation to acquire scarce nutrient resources from the soil solution [1]. Rapid root elongation allows foraging for water and ions in the soil, while active root branching at sites of locally high nutrient concentrations enhances nutrient uptake [2]. Despite sustained interest in root architecture regulation (the rate of root elongation and branching), many mechanisms are still not fully understood.

Increased biomass allocation to root growth is another common response to nitrogen (N) and phosphorus (P) deficits [3,4], with each element inducing some specific changes in root architecture. While low N primarily stimulates root elongation [5], P deficit increased root branching [6,7,8,9]. Effects of P-starvation on root elongation are rather contradictory. While P starvation decreased the length of *Arabidopsis* roots [10], longer roots were generated in cereal crops such as maize (*Zea mays*) [11], barley (*Hordeum vulgare)* [12], and rice (*Oryza sativa*) [10]. There is increasing interest by plant breeders in identifying QTLs (Quantitative Trait Locus) regulating the root architectural responses of cereal crops to nutrient deficits [13] with co-location of root architecture and hormone biosynthesis/metabolism QTLs [14].

Environmentally-mediated changes in phytohormone concentrations and sensitivity are suggested to regulate root architecture [15]. N re-supply increases cytokinin (CK) levels [16], with decreased endogenous CK concentrations under nutrient scarcity believed to enhance root growth relative to the shoot [17]. These hormones inhibit root growth by promoting the rate of meristematic cell differentiation and thereby decreasing root-meristem size and the rate of root growth [18]. Cytokinins repress cell division, exhausting the quiescent center [19]. While nitrates induce root *IPT* (isopentenyl transferase responsible for de novo CK synthesis) gene expression [16,20,21,22], the effects of phosphate starvation on CK levels have received little attention.

Although numerous reviews mentioned the importance of CKs for plant adaptation to phosphate starvation [9,23,24,25,26,27,28], relatively few experimental studies have been performed with mechanisms regulating shoot and root CK levels. Although P deficits decreased tissue CK concentrations [29,30,31], measurements often used whole seedlings or organs since the detection methods for CKs used then had relatively low sensitivity. Although P-starvation decreased expression of the *IPT3*-gene in both roots and shoots of Arabidopsis [32], tissue CK concentrations have only recently been measured [33,34,35].

Decreased root and shoot CK concentrations occurred simultaneously with changes in other plant hormones, suggesting a more complex plant growth regulation.

Typically, several hormones interact to regulate root growth under nutrient deficits. When wheat plants were grown in dilute (1/100th strength) Hoagland’s solution, increased root ABA concentrations activated cytokinin-oxidase, thereby decreasing CK concentrations and increasing root-to-shoot ratio [17]. Moreover, all members of the *IPS* (“induced by phosphate starvation”) gene family are controlled by both CKs and ABA, suggesting considerable crosstalk between these hormones [25]. However, the effects of ABA on CK levels in P-starved plants have not been studied.

ABA is suggested to activate root growth under P starvation since ABA-treated and P-deprived plants have similar growth patterns, such as increased root-to-shoot ratio [24]. Nevertheless, some reports show increased ABA concentrations in P-starved plants [33,35], while others report a decline [36,37] or no difference in ABA deposition into leaves of P-deficient and control plants [38]. Expression of the *PHR1* (PHOSPHATE STARVATION RESPONSE) gene inducible by phosphate starvation was diminished in the ABA deficient *Arabidopsis* mutant *aba*2-4, thereby confirming that this hormone is involved in responses to P deficit [39]. However, wild-type and ABA-deficient or ABA-insensitive *Arabidopsis* plants (*aba-1* and *abi2-1* respectively) all showed increased root-to-shoot ratio in response to P starvation [40], suggesting that other hormones were involved in this adaptive response. To our knowledge, ABA deficient monocot mutants have not previously been used to study hormone interactions in plants experiencing P deficit.

Cytokinin-auxin interactions are likely important under P deficit. Low P availability mimics auxin’s action in promoting lateral root development in *Arabidopsis* [41], suggesting that auxins are involved in the phosphate starvation response. Auxin accumulates in the tips of primary roots in the early stages of the P starvation response [42]. P deficiency increased the transcript levels of auxin-responsive genes (*AUX1*, *AXR1*, and *AXR2*), indicating activation of the auxin response pathway in P-starved plants. In the *aba2-4 Arabidopsis* mutant, the transcript levels of these genes did not increase, suggesting that ABA synthesis is to some extent required to induce auxin-responsive genes when plants are P-starved [39]. However, whether auxin accumulation in P-starved plants is ABA-dependent is unknown.

Our objective was to determine the role of multiple hormone interactions in regulating root growth responses to P deficit and consider the role of ABA status (bulk root and in root tips) in determining local (root) and long-distance (shoot) responses to P deficit. To study hormone interactions in plants exposed to P starvation, the ABA-deficient barley mutant *Az34* [43] and its wild-type (WT) were grown in P-deprived and P-replete conditions and endogenous hormone (ABA, the auxin indoleacetic acid (IAA) and zeatin-type cytokinins) concentrations measured in both the bulk roots and root tips along with plant growth responses. Mechanisms regulating endogenous CK (cytokinin oxidase enzyme activity, *HvIPT1* gene expression, whose abundance was highest in the root tips of barley seedlings) were evaluated in root tips. We hypothesized that limited ABA accumulation in the mutant compromised P-adaptive responses in the roots.

## 2. Results

At the beginning of our research, we compared growth responses to P-starvation in ABA-deficient barley mutant *Az34* and its wild-type (WT) plants.

**Figure 1:**
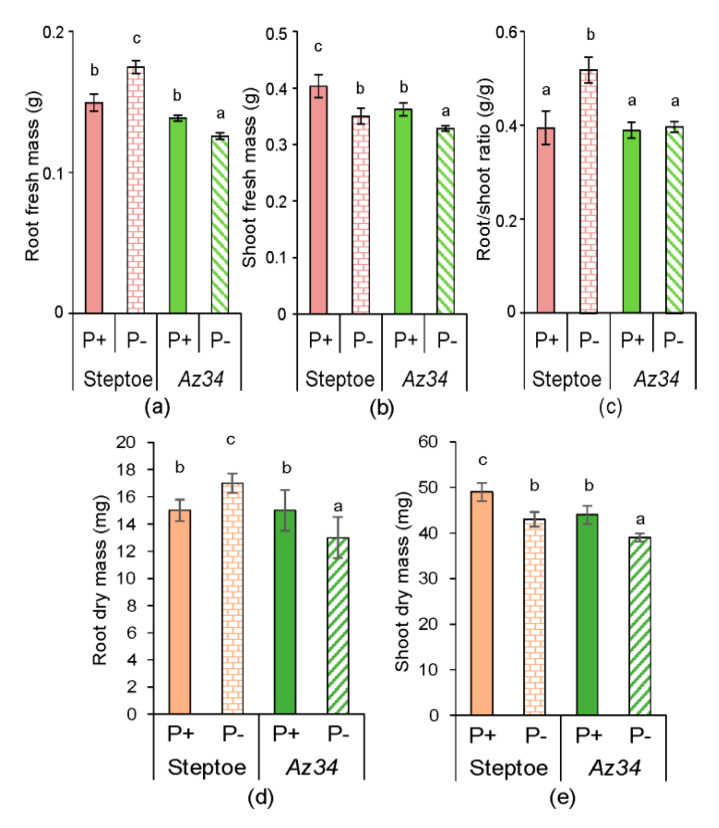
Root (**a**,**d**) and shoot (**b**,**e**) fresh (**a**,**b**) and dry (**d**,**e**) mass and root/shoot fresh mass ratio (**c**) of 7-days-old of WT (cv. Steptoe) and Az34 plants grown for 4 days on nutrient solutions with (P+) or without (P−) phosphate. Bars are means ± S.E. of *n* = 20, with significant (*p* ≤ 0.05) differences between all genotype/treatment combinations marked with different letters (ANOVA, LSD).

There were no genotypic differences in either fresh (Figure 1a) or dry (Figure 1d) root mass under P-replete conditions, but both the fresh and dry shoot mass of WT (cv. Steptoe) plants was 10% higher than in *Az34* plants (Figure 1b,e). P starvation increased root mass and decreased shoot mass of the WT plants (Figure 1a,b) while both root and shoot mass were decreased in *Az34*. As a result, P starvation increased the root/shoot mass ratio in the WT plants but did not affect root/shoot mass ratio in *Az34* plants (Figure 1c).

Thus, shoot and root responses to P starvation differed between genotypes, with both showing shoot growth inhibition but root growth promotion only occurring in WT plants. The similarity in the shoot growth response of *Az34* and WT plants was supported by the insignificance of interaction between genotype × P level, while the difference in root response of the genotypes is indicated by significant genotype × P level interaction (Table S1).

**Figure 2:**
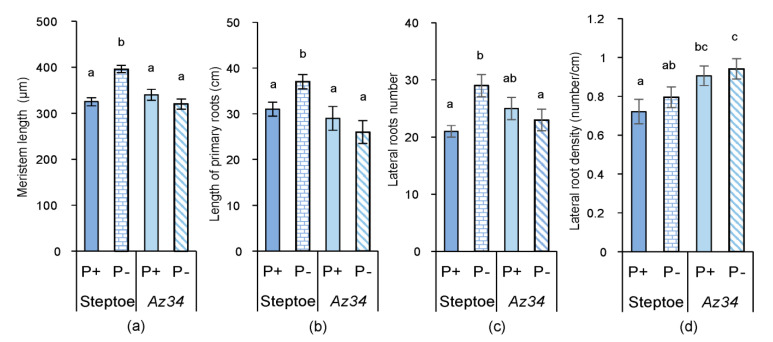
Root characteristics of WT (cv. Steptoe) and *Az34* plants grown on nutrient solutions with (P+) or without (P−) phosphate: meristem size measured in 4-days old plants 1 day after the start of P-treatment (**a**), the total length of all primary roots (**b**), number of lateral roots (**c**), lateral root density (**d**) measured in 7-days-old plants 4 days after the start of P-treatment. Bars are means ± S.E. of *n* = 20, with significant (*p* ≤ 0.05) differences between all genotype/treatment combinations marked with different letters (ANOVA, LSD).

While there were no genotypic differences in length of primary roots or lateral root number under P-replete conditions, the lateral root density (ratio of the number of lateral roots and root length) was 25% higher in *Az34* plants (Figure 2) resulting from the division of a slightly greater number of laterals by slightly smaller root length.

P starvation accelerated primary root elongation by about 20% and increased root branching by 38% in the WT plants but had no significant effect on primary root length or lateral root number of the *Az34* mutant (Figure 2b,c). The root length correlated with the size of root meristem (r = 0.92), suggesting that increased root length detected in P-starved Steptoe plants was due to the increase in cell division resulting in the increased size of the meristem zone (Figure 2a). Lateral root density was not affected by P treatment in either genotype (Figure 2d). Thus, the effect of P level on root mass, length, and branching depended on genotype (significant genotype × P level interactions—Tables S1 and S2).

Treatment of *Az34* plants with ABA resulted in a 16% increase in root mass of P-starved (from 0.126 ± 0.002 to 0.146 ± 0.002 g) and an 18% increase in their root length (from 26 ± 2 to 31 ± 2 cm), mimicking the effect of P-starvation on Steptoe plants.

**Figure 3:**
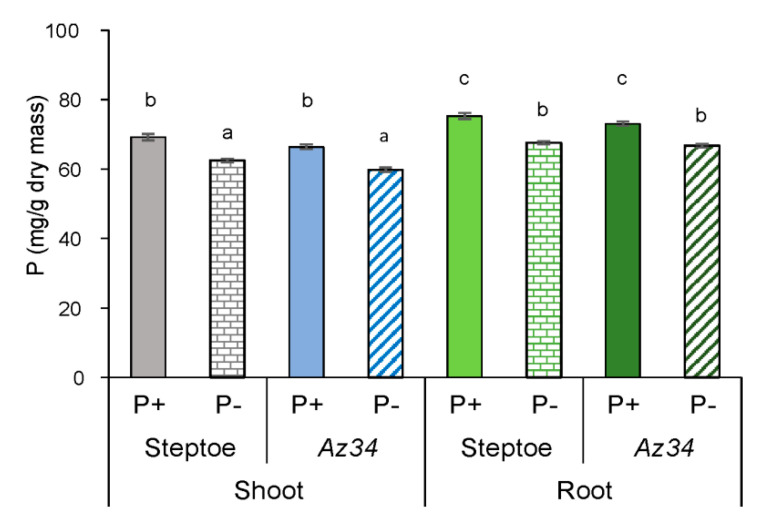
P concentration (mg/g dry mass) in shoots and roots of 4-days-old WT (cv. Steptoe) and *Az34* plants grown for 4 days on nutrient solutions with (P+) or without (P−) phosphate. Means (*n* = 6), with significant (*p* ≤ 0.05) differences between all genotype/treatment combinations are marked with different letters (ANOVA, LSD).

Tissue P concentrations were similar in both genotypes in P-replete conditions and decreased by about 10% in roots and shoots of both genotypes under P deficit showing a similar extent of P deficit in both genotypes (Figure 3). Thus, differences in root growth response between genotypes could not be attributed to tissue nutrient relations differences.

**Figure 4:**
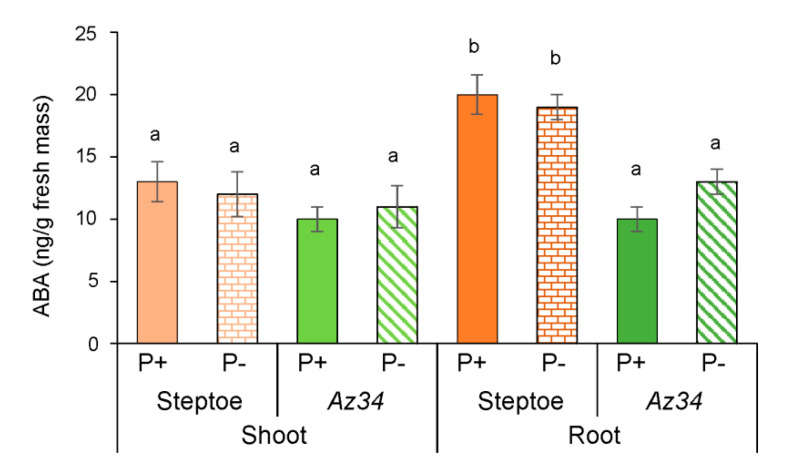
ABA concentration (calculated per g fresh mass) in shoots and roots of 4-days-old barley plants (cv. Steptoe and *Az34*) grown for 1 day on nutrient solutions with (P+) or without (P−) phosphate. Bars are means ± S.E. of *n* = 9, with significant (*p* ≤ 0.05) differences between all genotype/treatment combinations marked with different letters (ANOVA, LSD).

Next, we measured hormone concentration in plants trying to relate them to plant growth responses. The shoot ABA concentration of the WT and *Az34* plants did not differ and was not responsive to P level (Figure 4, Table S3). Although the root ABA concentration of WT plants was approximately double that of *Az34* plants, again, it did not depend on the phosphate level (Figure 4). These genotypic differences in root ABA concentration did not co-occur with root system morphology differences under P-replete conditions (cf. Figure 1, Figure 2 and Figure 4). Nevertheless, they were associated with genotypic differences in root ABA adaptations to P deficit as indicated by significant genotype × P level interaction (Table S3).

**Figure 5:**
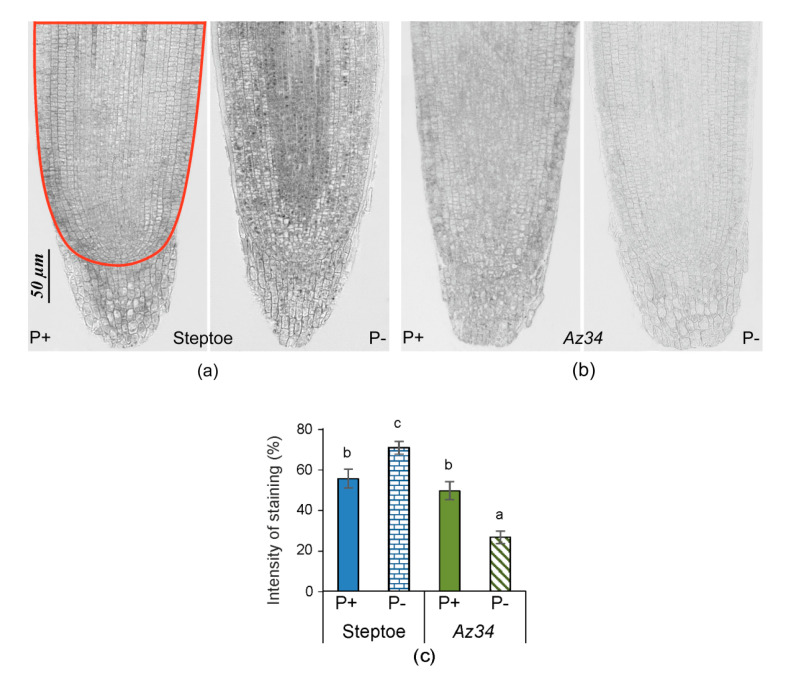
Immunolocalization of ABA in root tips of 4-days-old barley seedlings (cv. Steptoe (**a**) and *Az34* (**b**)) grown for 1 day on the nutrient solutions with (P+) or without (P−) phosphate. Scale bars 50µm. The staining intensity on all sections was evaluated in the area marked with a colored line in Figure 5a. Diagram (**c**) presents the results of a semi-quantitative assay of the intensity of staining of the tips of the root of Steptoe and *Az34* plants obtained with the help of the ImageJ program [44]. Images were taken from 15 independent sections per genotype or treatment. The intensity of staining was expressed in arbitrary units, with maximal staining taken as 100% and minimal as 0 with different letters above the bars indicating significant (*p* < 0.05) differences according to ANOVA (LSD).

Under P-replete conditions (Figure 5), there were no significant genotypic differences in root tip staining for ABA (consistent with the bulk root ABA data and (Table S3). Unlike bulk root ABA concentration, P starvation intensified the immunostaining for ABA in root tips of WT plants, but the opposite response occurred in *Az34* (Figure 5), as indicated by significant genotype × P level interaction (Table S3). Thus, P starvation resulted in significant genotypic differences in root tip ABA concentration (Figure 5).

**Figure 6:**
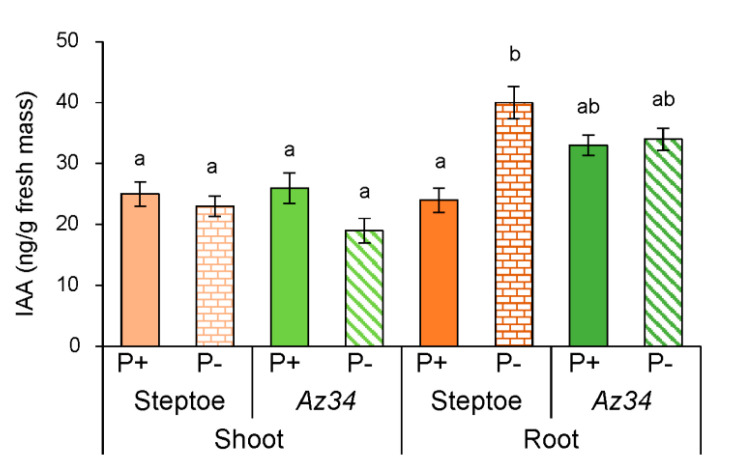
IAA concentration (calculated per g fresh mass) in shoots and roots of 4-days-old barley plants (cv. Steptoe and *Az34*) grown for 1 day on nutrient solutions with (P+) or without (P−) phosphate. Bars are means ± S.E. of *n* = 9, with significant (*p* ≤ 0.05) differences between all genotype/treatment combinations marked with different letters (ANOVA, LSD).

Shoot and root IAA concentrations of the WT and *Az34* plants did not significantly differ irrespective of P level. While P starvation increased root IAA concentration by 40% in WT roots, the root IAA concentration of *Az34* was not influenced by P availability. Root IAA concentrations of *Az34* were intermediate between the low values of WT plants grown in P-replete conditions and the higher values of WT plants grown in P-starved conditions (Figure 6). The difference in the changes in root IAA concentration induced by P level in WT and *Az34* plants was indicated by a significant genotype × P level interaction (Table S4).

**Figure 7:**
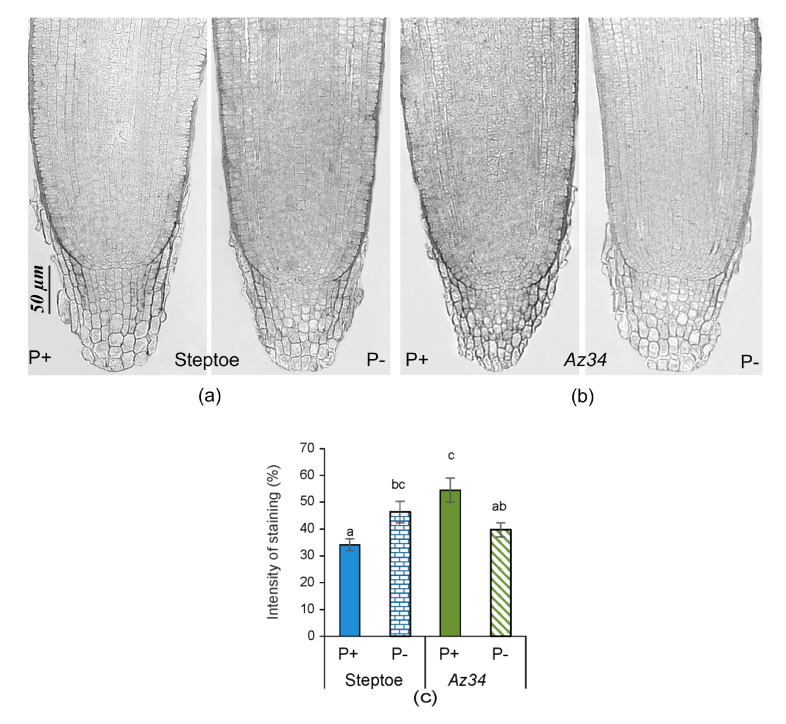
Immunolocalization of IAA in root tips of 4-days-old barley seedlings (cv. Steptoe (**a**) and *Az34* (**b**)) grown for 1 day on the nutrient solutions with (P+) or without (P−) phosphate. Scale bars 50 µm. The intensity of staining was evaluated in the area shown for Figure 5a. Diagram (**c**) presents the results of a semi-quantitative assay of the intensity of staining of the tips of the root of Steptoe and *Az34* plants obtained with the help of the ImageJ program [44]. Images were taken from 15 independent sections per genotype or treatment. The intensity of staining was expressed in arbitrary units, with maximal staining taken as 100% and minimal as 0 with different letters above the bars indicating significant (*p* < 0.05) differences according to ANOVA (LSD).

Under P-replete conditions, *Az34* had higher root tip staining for IAA (Figure 7) (inconsistent with the bulk root IAA data, which showed no genotypic differences (Figure 6)). Again, P starvation resulted in different responses between the genotypes, with immunostaining for IAA increasing in root tips of WT plants but decreasing in *Az34* (Figure 7), as indicated by significant genotype × P level interaction (Table S4). Thus, P starvation tended to minimize genotypic differences in root tip IAA concentration.

**Figure 8:**
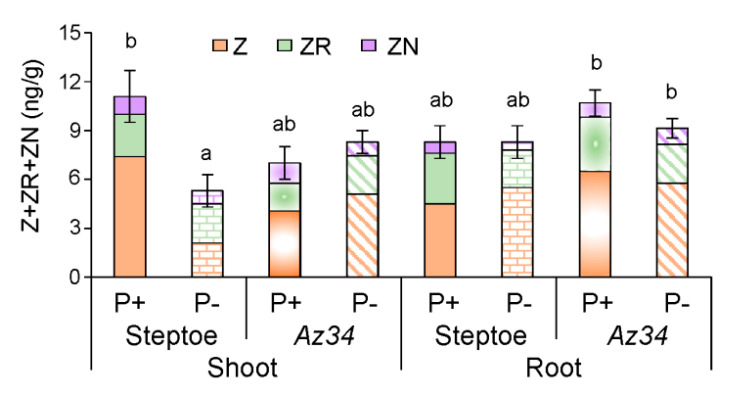
Sum of cytokinins (zeatin (Z), its riboside (ZR) and nucleotide (ZN)) concentration (calculated per g fresh mass) in shoots and roots of 4-days-old barley plants (cv. Steptoe and *Az34*) grown for 1 day on nutrient solutions with (P+) or without (P−) phosphate. Bars are means ± S.E. of *n* = 9 for the sum of zeatin derivatives, with significant (*p* ≤ 0.05) differences between all genotype/treatment combinations marked with different letters (ANOVA, LSD).

Zeatin was the most abundant of the different cytokinin forms measured, and its concentrations were summed along with ZR and ZN to calculate total CK concentrations (Figure 8). No significant difference was found between *Az34* and Steptoe in the CK concentration of the shoot or root at any P levels. P starvation approximately halved shoot CK concentrations in WT plants, the effect being most pronounced in the case of free zeatin (Figure 8), while the ABA-deficient *Az34* mutant showed no change in shoot CK concentration (Figure 8). The effect of P level on shoot CK concentrations depended on the genotype supported by two-way ANOVA (significant genotype × P level interaction—Table S5). In contrast, bulk root CK concentration did not change in both genotypes (Figure 8). The changes in each of the zeatin derivates followed regularities detected for the sum of cytokinins. The trend of the increase in zeatin and decline in its riboside induced by P-starvation in Steptoe was not statistically significant.

**Figure 9:**
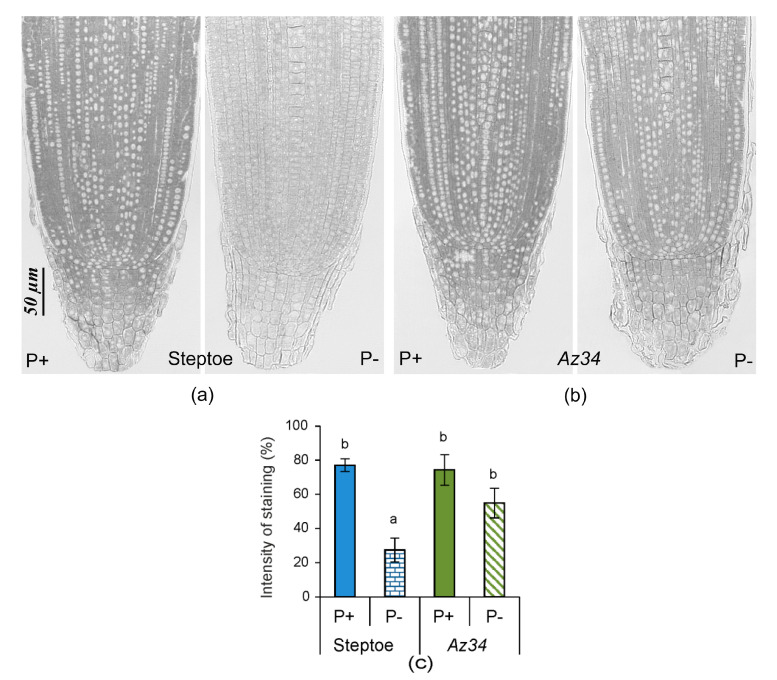
Immunolocalization of free zeatin in root tips of 4-days-old barley seedlings (cv. Steptoe (**a**) and *Az34* (**b**)) grown for 1 day on the nutrient solutions with (P+) or without (P−) phosphate. Scale bars 50 µm. The intensity of staining was evaluated in the area shown for Figure 5a. Diagram (**c**) presents the results of a semi-quantitative assay of the intensity of staining of the tips of the root of Steptoe and *Az34* plants obtained with the help of the ImageJ program [44]. Images were taken from 15 independent sections per genotype or treatment. The intensity of staining was expressed in arbitrary units, with maximal staining taken as 100% and minimal as 0 with different letters above the bars indicating significant (*p* < 0.05) differences according to ANOVA (LSD).

Under P-replete conditions, there were no significant genotypic differences in root tip staining for zeatin. P starvation decreased staining for CK in the WT root tips but had no significant effect in *Az34* (Figure 9). Dependence of P effect on genotype was supported by a significant genotype × P level interaction (Table S5). Thus, immunolocalization revealed genotypic differences under P starvation that were not observed in (bulk root) CK concentrations.

**Figure 10:**
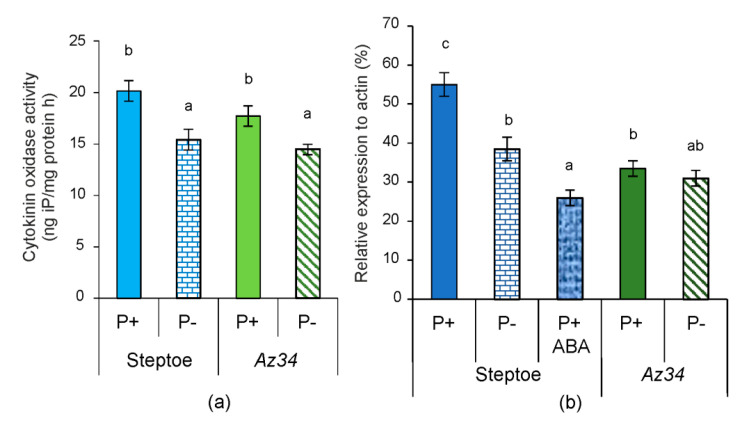
Cytokinin oxidase activity (**a**) and *HvIPT1* transcript abundance (**b**) in root tips of 4-days-old barley seedlings (Steptoe and *Az34* genotypes) grown for 1 day on the nutrient solutions with (P+) or without (P−) phosphate. ABA was added to the nutrient solution one day prior to sampling (P+ ABA). Data are the mean of five determinations, with different letters above the bars indicating significant (*p* < 0.05) differences according to ANOVA (LSD).

P starvation could alter the levels of root tip CKs by either modifying cytokinin synthesis or cytokinin catabolism. Root tip cytokinin oxidase activity was measured to evaluate the impact of cytokinin metabolism on their level, but no significant difference was detected between genotypes irrespective of P level (Figure 10a). Since P-starvation decreased cytokinin content, we expected an increase in cytokinin oxidase activity, which could be the cause of cytokinin decline. Contrary to expectations, P starvation decreased root tip cytokinin oxidase activity by about 25% in both genotypes (Figure 10a). The similarity in both genotypes’ responses to P level was supported by insignificant genotype × P level interaction (Table S6). Thus, there were no genotypic differences in root tip cytokinin oxidase activity or response to P starvation.

Since cytokinin catabolism changes could not account for root tip responses to P starvation, expression of the *HvIPT1* gene (responsible for de novo cytokinin synthesis) was analyzed. This gene of the IPT family was chosen since its abundance was highest in barley seedlings’ root tips. In P-replete conditions, *HvIPT1* gene expression was 30% higher in WT’s root tips than in *Az*34 plants (Figure 10b). P starvation significantly decreased the level of the *HvIPT1* transcript in the WT plants but induced no response in *Az34* plants. The difference in response of *Az34* and Steptoe to P level was supported by a significant genotype × P interaction (Table S6). Applying exogenous ABA to WT plants also lowered this gene’s transcript level (Figure 10b). While *HvIPT1* expression increased with bulk root ABA concentration in P-replete conditions (when comparing the two genotypes), while additional root tip ABA accumulation decreased *HvIPT1* expression.

## 3. Discussion

P starvation decreased shoot mass similarly in both WT and ABA deficient mutant plants (Figure 1), indicating that ABA was not involved in regulating shoot growth responses to P starvation. Alternatively, previous experiments with other barley cultivars attributed shoot growth inhibition of P-starved plants to decreased shoot CK concentrations [33], as these hormones maintain shoot growth [45] by stimulating cell division [46] and elongation [47]. Although shoot CK concentrations decreased in P-starved WT plants (Figure 8), they did not change in *Az34*, while P starvation decreased both genotypes’ shoot growth. Therefore, inhibition of shoot growth cannot be attributed to decreased CK concentrations. Shortage of the phosphorus necessary to maintain shoot growth (tissue concentrations declined by 10% in both genotypes) may account for shoot growth inhibition.

Since P starvation affected each genotype’s root growth differently (significant genotype × P level interactions—Table S1) despite similar root P concentrations, alternative (hormone interaction) explanations were sought. WT plants increased their root mass and the total length of all primary roots following P-starvation (Figure 1 and Figure 2), whereas root mass decreased, and root length did not change in the ABA-deficient *Az34*. These results showed that capacity for ABA accumulation in the root tips (characteristic of WT, but absent in *Az34*) is required for root growth adaptation to P starvation manifested in relative activation of root growth (increased root-to-shoot ratio detected in P-starved WT, but not in *Az34*). In contrast, the root-to-shoot ratio of both WT and ABA-deficient *Arabidopsis* mutants increased under phosphorus-deficient conditions [40], possibly since sucrose (present in the *Arabidopsis* growth medium) modifies root growth responses to P starvation [48]. ABA-dependent differences in barley’s root growth response to P starvation (Figure 1 and Figure 2) occurred even though bulk root ABA concentration did not change in either genotype in response to P starvation (Figure 4). Nevertheless, root tip ABA concentration increased in WT plants and declined in *Az34* plants (Figure 5). This could accelerate the root growth of WT plants since root elongation occurs in the root tips.

Similarly, mineral nutrients’ dilution increased ABA concentrations in root tips but not in whole roots in WT plants [49]. Root apical ABA accumulation maintains root growth in plants under water [50] and mineral nutrient [49] deficits and osmotic stress [51]. Therefore, ABA regulates root growth responses to different stresses, including P deficit. Alongside possible direct effects of ABA, it can also regulate root growth by affecting root CK levels, as these hormones inhibit root growth [47]. ABA’s importance for the control of cytokinin level in the root tips of P-starved plants is supported by the absence of cytokinin response in ABA deficient *Az*34. Although bulk root CK concentration did not change in either genotype in response to P starvation (Figure 8), root tip CK concentrations (detected with immunostaining, Figure 9) decreased in WT plants, but there was no significant effect in *Az34* roots. The decreases in root tip concentration in WT plants were likely to be responsible for increasing the size of root zone meristem (Figure 2a). Our results are in accordance with other reports showing the increased size of the meristem zone in roots of *ipt* mutant plants of *Arabidopsis*, in which the endogenous CK level was lower than in wild-type roots (Cytokinins regulate root growth through its action on meristematic cell proliferation but not on the transition to differentiation [52]. The correlation of root meristem size with the root length detected in the present experiments is in accordance with other reports [53,54].

The decline in shoot cytokinin concentration was attributed to (CKX) activation in shoots when wheat plants were exposed to nutrient dilution [17]. However, P starvation decreased root CKX activity of both genotypes (Figure 10a), and alternative mechanisms must account for decreased CK concentrations. Our results show that the decline in P-starved WT plants’ root tip cytokinins is likely to be due to decreased expression of isopentenyltransferase gene. The importance of isopentenyltransferase (IPT) is because it is responsible for the rate-limiting step of cytokinin biosynthesis [55]. Accumulation of CKs was greatly attenuated in an *ipt* mutant of *Arabidopsis* [16]. Dependence of the decline in the ABA gene expression is supported by our observations showing that P starvation down-regulated root tip *HvIPT1* gene expression in WT plants but had no significant effect in ABA deficient *Az34* (Figure 10b). The importance of ABA accumulation for down-regulation of *HvIPT1* gene expression is confirmed by our data showing that exogenous ABA down-regulated root tip *HvIPT1* gene expression in P-replete WT plants (Figure 10b). P starvation of rice (*Oryza sativa*) seedlings downregulated genes for CK signaling components (*OsRR6* and *OsRR9/10*), suggesting that P starvation affects both CK levels and downstream signaling [56].

Genotypic differences in root hormone concentration were also associated with differences in lateral root development, as P starvation increased the lateral root number of WT plants but had no effect in *Az34* (Figure 2). P starvation increased both bulk root and root tip auxin concentrations in WT plants but had no effect (bulk root) or decreased (root tip) auxin concentration in *Az34*. Increased auxin concentration detected by us in the roots of WT plants is in accordance with reports, where P deficiency increased the transcript levels of auxin-responsive genes (*AUX1*, *AXR1,* and *AXR2*) [39], which may serve as an indirect indication of an increase in auxin concentration in response to P deficiency. Since auxins stimulate root branching [57], increased bulk root IAA concentrations may be related to the increased number of lateral roots in WT plants. P starvation usually increases root branching in *Arabidopsis* [7], thereby enhancing root capacity for phosphate uptake [9], although decreased root branching occurred in wheat [58]. Since *Az34* roots failed to accumulate both ABA and auxin following P starvation (independent of whether measurements were made in the bulk roots or root tips—Figure 4, Figure 5, Figure 6 and Figure 7), our results confirm the impotence of the interaction of these two hormones in regulating root branching, which is in accordance with other reports [59,60,61].

ABA has been shown to influence root branching in an opposite way depending on the stage of lateral root formation [62,63]. ABA promotes new lateral root primordia by stimulating their initiation in the root tips, while elongation of the lateral root and lateral root emergence are repressed by ABA distantly from the root tip. In accordance with this information, the accumulation of ABA in the root tips, but not in the whole roots detected in the present experiments is likely to promote root branching. ABA’s effects on lateral root development are highly dependent on the growth medium, with the ABA-deficient tomato mutants *notabilis* and *flacca* showing increased lateral root development when grown in vitro [64], but fewer lateral roots when grown in soil (*notabilis*—[65]. This discrepancy in ABA action may be due to its opposing effects on auxin level: ABA can either decrease auxin content by activating IAA conjugation [66] or enable its accumulation by activating IAA synthesis [67]. The latter mechanism operates in our experiments since P-starved WT plants’ root tips accumulated both ABA and IAA in parallel, while P-starved *Az34* plants showed decreases in both ABA and IAA (cf. Figure 5 and Figure 7). Since auxin-induced initiation of root primordia starts with anticlinal divisions in the pericycle occurring in the root tips [68], parallel accumulation of IAA and ABA in the root tips of WT plants (Figure 5 and Figure 7) and the opposite response in *Az34* confirms that ABA and auxin interact to stimulate root branching under P starvation. Bulk root IAA accumulation in P-starved WT plants (Figure 6) provides an additional stimulus for the emergence of lateral roots.

## 4. Materials and Methods

### 4.1. Plant Growth Conditions and Treatments

Experiments used wild-type (WT) barley plants (*Hordeum vulgare* L. cv. Steptoe) and its ABA deficient mutant *Az34*. Seeds were allowed to germinate in darkness, floating in water in sealed and tied together glass tubes for 3 days at 24 °C. Three-days old seedlings were transferred to modified 0.1 strength Hoagland–Arnon nutrient medium (0.5 mM KNO_3_, 0.5 mM Ca(NO_3_)_2_, 0.1 mM KH_2_PO_4_, 0.1 mM MgSO_4_, 0.5 mM CaSO_4_), where KH_2_PO_4_ was either omitted (P−) or substituted with NaH_2_PO_4_, (P+) and seedlings were grown at a 14-h photoperiod and an irradiance of 400 µmol m^−2^ s^−1^ from mercury-arc and sodium vapor lamps. Preliminary experiments showed that substituting KH_2_PO_4_ for NaH_2_PO_4_ did not influence plant growth [33]. Simultaneously, with the start of the P-treatment, ABA was added to some plants’ nutrient solution to yield a final concentration of 2 µM. One day after imposing the P- treatment, the shoots and roots of 4-days-old plants were sampled for hormone, phosphate, and PCR analyses, and root sections were taken for immunolocalization studies and measurement of meristem length. Four days after imposing the treatments, shoot and root fresh and dry mass, root length, and the number of lateral roots was measured in 20 7-days-old plants per genotype and treatments. For measuring dry mass, the shoot and roots were weighed after their drying at 70 °C until they reached constant mass. Experiments were repeated three times with similar results.

### 4.2. Hormone Analyses and Immunolocalization

Shoots and roots of 4 plants were sampled for hormone extraction (number of replicates, *n* = 9). Hormones were extracted from homogenized shoots and roots of barley plants with 80% ethanol overnight at 4 °C. Cytokinins and acidic hormones (ABA and IAA) were extracted in different ways from aliquots of aqueous residue described by Vysotskaya et al. [17]. In short, CKs were concentrated on a C18 column, washed with water, eluted with 80% ethanol, and separated using thin-layer chromatography on silica gel plates in a mixture of 2-butan-ol, ammonium, and water (6:1:2 *v*/*v*). Eluates from the zones corresponding to the position of cytokinin standards were immunoassayed with the help of an antiserum raised against zeatin riboside (ZR), shown to have high specificity to zeatin derivatives [69]. Cross-reactivity of anti-ZR serum to derivatives of other cytokinin bases (dihydrozeatin and isopentenyladenine) is low. This method has proven to be reliable by testing its results against a physico-chemical assay [70]. ABA and the auxin IAA were partitioned with diethyl ether from the aqueous residue after diluting with distilled water and acidification with HCl to pH 2.5. The hormones were then transferred from the organic phase into a solution of NaHCO_3_, re-extracted from the acidified aqueous phase with diethyl ether, and immunoassayed after methylation using antibodies to ABA and IAA [49]. Reducing the amount of extractant at each stage and re-extraction increased hormone recovery’s selectivity [71].

For immunolocalization of hormones, they were conjugated to proteins of the cytoplasm to prevent them from washing out during the dehydration process. Specifically, free cytokinin bases in tissues were fixed in a mixture 4% paraformaldehyde and 0.1% glutaradehyde, while ABA was fixed in carbodiimide, as described by Kudoyarova et al. [70] and Sharipova et al. [44], respectively. After washing with 0.1 M phosphate buffer, tissues were dehydrated in a series of ethanol dilutions. After this, the tissues were embedded in methylacrylate resin JB-4 (Electron Microscopy Sciences (Hatfield, PA, USA)). Immunolocalization of hormones was carried out using antisera against either ABA [41] or zeatin riboside (ZR) [70], depending on the type of fixed hormone. In short, diluted rabbit anti-ABA or anti-ZR sera were placed on the sections. Gelatin (0.2%) was added to the solution to prevent non-specific binding. After the sections were incubated in a humid chamber for 2 h and then washed with phosphate buffer containing Tween 20, they were treated with goat anti-rabbit immunoglobulins labeled with colloid gold. After incubation and washing, the sections were fixed in glutaraldehyde and incubated with silver enhancer. Antibodies raised against ZR recognized not only ZR but also free zeatin. Since the procedure of tissue fixation enabled conjugation of free bases and not their ribosides [72], immunostaining with anti-ZR serum is interpreted as visualization of zeatin. Earlier specificity and reliability of immunostaining was confirmed in experiments, where increased immunostaining was detected in the plants treated with exogenous hormones [44,70] or in transgenic plants with induced expression of *ipt* gene controlling cytokinin synthesis [73] (positive control). Non-immune rabbit serum was used as a control, and the absence of immunostaining when anti-ZR serum or anti-ABA serum was substituted with the non-immune serum confirmed the reliability of the technique. Images for immunolocalization of each hormone were taken from 15 independent sections per genotype or treatment. Figures present images of the meristem zone, where no significant increase in cell length was detected. Thus, immunolocalization revealed hormone content mostly in the meristem zone. For meristem length measurements, the border between meristem and elongation zone was defined by the first elongated cortex cell.

Cytokinin oxidase activity was determined as described previously [17,74]. In short, imidazole-buffer homogenate of root tips 3–5 mm long was centrifuged. A saturated solution of (NH_4_)_2_SO_4_ was added to the supernatant, centrifuged, and the pellet resuspended. Synthetic iPA (N6-isopentenyladenosine) was added to the suspension as a substrate, and the mixture was incubated for 2 h. An immunoassay, using antibodies raised against iPA, determined the amount of iPA lost due to degradation.

### 4.3. RNA Extraction and Analysis of Abundance of HvIPT1 mRNA

Total RNA was isolated from the root tips 3–5 mm long with Trizol reagent. The first strand of cDNA was created using oligo (dT) primer and M-MuLV-reverse transcriptase (New England Biolabs, Ipswich, MA, USA). The following primers were used for quantitative analysis of *HvIPT1* (AK250176.1) barley gene expression (shown in preliminary experiments to be highly abundant in the root tips of barley seedlings: 5′-GCAGGCCATTGGGGTTCGTGA-3′ and 5′-CTCGCCCTTGCTTGTGTTGTTCC-3′ (size of amplicon is 479 bp). Real-time PCR was performed in the presence of SYBR Green I intercalating dye in Rotor-GeneTM 6000 thermocycler (Corbett Research, Australia). The PCR was performed with 30 cycles at 94 °C for 30 s, 57 °C for 30 s, and 72 °C for 1 min. mRNA of actin protein was used as the standard for calculations, and its expression level was taken as 100% [75]. RT-PCR of the actin gene of barley (MK034133) was performed using primer pair 5′-TGCGTACGTTGCCCTTGATTATGA-3′ and 5′-GCCACCACTGAGCACGATGTTTC-3′ (size of amplicon is 259 bp).

### 4.4. Determination of Phosphorus in Roots and Leaves of Barley Plants

Total P content was determined in dry roots and shoots after digestion with H_2_SO_4_ and KClO_4_. Determination of phosphates in the root digest was carried out with the molybdenum-blue method using stannous chloride as the reducing agent, as described in Reference [76].

### 4.5. Statistical Analysis

Two-way analysis of variance (ANOVA) determined the effects of genotype, P treatment and their interaction. One-way ANOVA was applied across different genotype/treatment combinations, with the least significance difference (LSD) test to discriminate means.

## 5. Conclusions

In summary, P starvation increased root elongation of WT barley plants by stimulating root tip ABA accumulation and decreasing root tip cytokinin concentrations. Decreased root tip cytokinin concentrations in P-starved WT plants were not due to the changes in root CKX activity but down-regulation of the *HvIPT1* gene. Furthermore, increased root branching was related to bulk root IAA accumulation. Both hormonal and root growth responses were not detected in the *Az34* mutant with a low ABA accumulation capacity. The absence of these effects in ABA deficient *Az34* mutant demonstrates that ABA accumulation capacity is important in regulating root branching and elongation following P starvation by affecting concentrations of IAA and cytokinins.

## Supplementary Materials

The following are available online at https://www.mdpi.com/2223-7747/9/12/1722/s1, Table S1: Analysis of variance of the shoot and root mass, root/shoot fresh mass ratio of WT and Az34 genotypes grown for 4 days on nutrient solutions with or without phosphate (P level). P values are presented for the effects of genotype, P level, and their interaction, Table S2: Analysis of variance total primary root length, lateral root number, and lateral root density of WT and *Az34* genotypes grown for 4 days on nutrient solutions with or without phosphate (P level). P values are presented for the effects of genotype, P level, and their interaction, Table S3: Analysis of variance of ABA concentrations of WT and *Az34* genotypes grown for 1 day on nutrient solutions with or without phosphate (P level). P values are presented for the effects of genotype, P level, and their interaction, Table S4: Analysis of variance of IAA concentrations of WT and *Az34* genotypes grown for 1 day on nutrient solutions with or without phosphate (P level). P values are presented for the effects of genotype, P level, and their interaction, Table S5: Analysis of variance of total concentrations of zeatin derivatives (free zeatin+ zeatin riboside+zeatin nucleotide) of WT and *Az34* genotypes grown for 1 day on nutrient solutions with or without phosphate (P level). P values are presented for the effects of genotype, P level, and their interaction. Table S6: Analysis of variance of cytokinin oxidase activity and *HvIPT1* transcript abundance in the root tips of Steptoe and *Az34* (genotypes) grown for 1 day on the nutrient solutions with or without phosphate (P level). P values are presented for the effects of genotype, P level, and their interaction.
