# Supplementary material for: Effects of Phosphate Shortage on Root Growth and Hormone Content of Barley Depend on Capacity of the Roots to Accumulate ABA
Source: Plants (Basel). 2020 Dec 7;9(12):1722. doi: 10.3390/plants9121722 (PMC7762276; doi:10.3390/plants9121722)
Supplement: Supplementary file 1 [file plants-09-01722-s001.pdf]

Table S1. Analysis of variance of shoot and root mass, root/shoot fresh mass ratio of WT and Az34 genotypes grown for 4 day on nutrient solutions with or without phosphate (P level). P values are presented for effects of genotype, P level and their interaction.

Effect or Interaction	Shoot Mass	Root Mass	Root/Shoot Ratio
Genotype	0.011	<0.001	0.014
P level	<0.001	0.10	0.011
Genotype x P level	0.70	<0.001	0.023

Table S2. Analysis of variance total primary root length, lateral root number and lateral root density of WT and *Az34* genotypes grown for 4 day on nutrient solutions with or without phosphate (P level). P values are presented for effects of genotype, P level and their interaction.

Effect or Interaction	Length of Primary Roots	Lateral Root Number	Lateral Root Density
Genotype	0.004	0.73	0.003
P level	0.49	0.068	0.31
Genotype x P level	0.026	0.003	0.72

Table S3. Analysis of variance of ABA concentrations of WT and *Az34 g*enotypes grown for 1 day on nutrient solutions with or without phosphate (P level). P values are presented for effects of genotype, P level and their interaction.

Effect or Interaction	Bulk Shoot	Bulk Root	Root Tips
Genotype	0.15	<0.001	<0.001
P level	0.76	0.49	0.43
Genotype x P level	0.62	0.05	<0.001

Table S4. Analysis of variance of IAA concentrations of WT and *Az34* genotypes grown for 1 day on nutrient solutions with or without phosphate (P level). P values are presented for effects of genotype, P level and their interaction.

Effect or Interaction	Bulk Shoot	Bulk Root	Root Tips
Genotype	0.56	0.44	0.08
P level	0.048	<0.001	0.75
Genotype x P level	0.31	0.002	0.001

Table S5. Analysis of variance of total concentrations of zeatin derivatives (free zeatin+ zeatin riboside+zeatin nucleotide) of WT and *Az34* genotypes grown for 1 day on nutrient solutions with or without phosphate (P level). P values are presented for effects of genotype, P level and their interaction.

Effect or Interaction	Bulk Shoot	Bulk Root	Root Tips
Genotype	0.54	0.14	0.046
P level	0.087	0.50	<0.001
Genotype x P level	0.019	0.35	0.015

Table S6. Analysis of variance of cytokinin oxidase activity and *HvIPT1* transcript abundance in the root tips of Steptoe and *Az34* (genotypes) grown for 1 day on the nutrient solutions with or without phosphate (P level). P values are presented for effects of genotype, P level and their interaction.

Effect or Interaction	Cytokinin Oxidase Activity	*HvIPT1* Transcript Abundance
Genotype	0.07	<0.001
P level	0.003	0.007
Genotype x P level	0.41	0.036
